# Dual-time-point FDG PET/CT imaging in prosthetic heart valve endocarditis

**DOI:** 10.1007/s12350-017-0902-3

**Published:** 2017-05-04

**Authors:** A. M. Scholtens, L. E. Swart, H. J. Verberne, R. P. J. Budde, M. G. E. H. Lam

**Affiliations:** 1 Department of Nuclear Medicine, Meander Medical Center, Maatweg 3, 3813TZ Amersfoort, The Netherlands; 2 Department of Radiology and Nuclear Medicine, Erasmus Medical Center, Rotterdam, The Netherlands; 3 Department of Radiology and Nuclear Medicine, Academic Medical Center, Amsterdam, The Netherlands; 4 Department of Radiology and Nuclear Medicine, University Medical Center Utrecht, Utrecht, The Netherlands

**Keywords:** FDG PET/CT, endocarditis, prosthetic heart valve

## Abstract

**Purpose:**

FDG PET/CT has been of increasing interest in the diagnostic workup of prosthetic heart valve endocarditis (PVE). Some reports advocate later imaging time points to improve the diagnostic accuracy for PVE. In this study, we compared standard and late FDG PET/CT images in patients with a clinical suspicion of PVE.

**Materials and Methods:**

Fourteen scans in 13 patients referred for FDG PET/CT for suspicion of PVE performed at standard (60 min post injection) and late (150 min post injection) time points were scored based on visual interpretation and semi-quantitatively with SUVmax and target-to-background ratio (TBR, defined as [SUVmax valve/SUVmean blood pool]). Final diagnosis was based on surgical findings in all cases of infection (*n* = 6) and unremarkable follow-up in all others (*n* = 8).

**Results:**

Late images were more prone to false positive interpretation for both visual and semi-quantitative analyses. Visual analysis of the standard images yielded 1 false negative and 1 false positive result. On the late images, no scans were false negative but 5 scans were false positive.

**Conclusion:**

Late FDG PET/CT imaging for PVE seems prone to false positive results. Therefore, late imaging should be interpreted with caution.

**Electronic supplementary material:**

The online version of this article (doi:10.1007/s12350-017-0902-3) contains supplementary material, which is available to authorized users.

## Introduction

Prosthetic heart valve (PHV) implantation is performed at an increasing rate, due to the prevalence of heart valve disease increasing in tandem with the growing aging population, with over 800,000 annual procedures estimated to be performed worldwide by the year 2050.^1^ Prosthetic heart valve (PHV) endocarditis (PVE) is a relatively uncommon complication, with an incidence of 0.3-1.0% per patient per year,^2^ but is associated with an alarmingly high mortality rate, especially when *Staphylococcus aureus* is the pathogen involved.^2^,^3^ PVE can be difficult to diagnose, with echocardiography unable to identify signs of the disease in up to 30% of cases.^4^,^5^

Computed tomography angiography (CTA) of the valve area has been shown to be of complementary value to clinical routine workup in suspected PVE,^6^ although it can be difficult to distinguish between non-infectious postoperative anatomical variation and infectious complications in select cases.

Fluorine-18 fluorodeoxyglucose positron emission tomography with CT-based attenuation correction (FDG PET/CT) is gaining momentum as a tool in the diagnosis of suspected PVE,^7^–^10^ due to its ability to image inflammation activity as opposed to the aforementioned modalities that image anatomy only. Recently, FDG PET/CT was added as a diagnostic modality in the guidelines of the European Society of Cardiology for the diagnosis and management of infectious endocarditis.^11^

However, the optimal imaging protocol for FDG PET/CT in PVE is still unclear, with preparatory protocols and timing of image acquisition still subjects of debate. In standard oncological FDG PET/CT protocols, images are acquired 60 minutes after injection of the tracer. However, for the detection of infection and inflammation, both earlier and later imaging have been proposed; the former based on the fast influx of glucose into inflammatory cells followed by efflux based on active glucose-6-phosphatase,^12^ the latter based on persistent influx in inflammation and further clearance of glucose from the blood pool leading to higher contrast between activity in infectious foci and background.^13^

Based on earlier reports, delayed imaging may be of additional value in diagnosing infection of cardiovascular implants.^13^,^14^ We performed both standard and delayed acquisitions of FDG PET/CT images in a number of patients referred for possible PVE under the assumption that the delayed images may improve diagnostic accuracy.

## Methods

Based on earlier reports,13,^14^ we added delayed acquisition at 150 minutes post injection of the radiotracer to our clinical protocol for FDG PET/CT for suspicion of PVE, in keeping with the innovation and development stages as described by the IDEAL framework.^15^ Thirteen patients with 14 scans referred for FDG PET/CT with suspected PVE in the University Medical Center Utrecht were imaged at standard and late time points after giving informed consent. The local ethical committee waived review of this study.

### FDG PET/CT

All patients were prepared according to our protocol for suppression of physiological myocardial glucose metabolism (low-carbohydrate diet for 12 hours followed by a 12-hour fast and 50 IU/kg heparin IV 15 minutes prior to FDG administration).^16^ After injection of 2 MBq/kg FDG via an antecubital vein, PET/CT images were acquired according to the standard protocol at approximately 60 minutes and additionally of the thorax at approximately 150 minutes post injection. All scans were performed on the same FDG PET/CT system (Biograph mCT, Siemens, Erlangen, Germany). Prior to the PET acquisitions, non-contrast CT images were obtained for attenuation correction (AC). PET images were obtained using 3D acquisition, field of view 216 mm, and 3 minutes per bed position scan time; low-dose CT acquisition for attenuation correction was obtained with a pitch of 1.0, slice thickness 10 mm, 120 kV, and 40 mAs. PET/CT data were reconstructed using ordered subset expectation maximization (OSEM) iterative reconstruction (Gaussian filter, 4 iterations, 21 subsets).

All images were read on commercially available software (Syngo.Via, Siemens, Erlangen, Germany). Volumes of Interest (VOIs) were placed to ascertain semi-quantitative measurements of glucose metabolism as follows:

- Automated growing VOI algorithm set to include pixels within 40% of maximum measured standardized uptake value (SUVmax) containing the PHV.
- VOI sphere within the lumen of the descending aorta (maximum possible size without including vessel wall).

Care was taken to exclude non-suppressed myocardial uptake and uptake in the aortic wall. From these VOIs, the SUVmax in the region of the PHV and the mean standardized uptake value (SUVmean) in the aortic blood pool were obtained. Target-to-background ratios (TBR) were calculated as SUVmax (PHV) divided by SUVmean (blood pool).^8^

Visual analysis was based on elevated uptake of FDG at or near the implanted PHV, taking into account normal variations and potential confounders.^17^ Elevated uptake at the PHV or adjacent structures that exceeded that of the surrounding blood pool and could not be ascribed to a normal variant was deemed suspect for PVE. Lesions on AC images were confirmed on non-AC images to rule out possible AC artifacts. Scoring was dichotomous into either no / unlikely PVE or likely / certain PVE.

### Diagnosis

The final diagnosis of infected versus uninfected PHV was based on surgical findings in all cases of infection and based on unremarkable follow-up after clinically rejected diagnosis of endocarditis (median 17.9 months, range 9.7-22.6 months) in all others.

### Analysis

Comparisons of means between the normally distributed semi-quantitative values for infected and uninfected PHVs were performed with paired Student’s *t*-test. Statistical analyses were performed on Statistical Package for Social Sciences (SPSS) software version 22. Additional ROC analysis was performed with MedCalc software version 16.4.3.

## Results

Scans were performed for suspected PVE of one mitral valve replacement, one pulmonic valve replacement, and 11 aortic valve replacements. In two patients, the aortic valve replacement was part of a Bentall graft of the aortic root and ascending aorta. Median time from implantation to FDG PET/CT was 654 days (range 21-4992 days). Two patients were scanned within the first six weeks after implantation. Standard images were acquired at a mean of 65 minutes post injection (range 56-80 minutes), and late images were acquired at a mean of 144 minutes post injection (range 120-195 minutes). The time between standard and late scans ranged from 57 to 115 minutes. One patient was scanned twice: once during antibiotic therapy to exclude other foci, and once when cessation of antibiotic therapy led to an increase in symptoms and the return of fever. Suppression of physiological myocardial glucose metabolism was acceptable in all but one patient. PVE was diagnosed and surgically confirmed in 6 out of 13 patients. Patient characteristics are listed in Table 1.

**Table 1:** Patient characteristics

Patient	Age	Valve		Days post implant	Implantation/complications	CRP	Leukocyte count	PVE
		Type	Location					
1	72	Mechanical	MV	4992	Uncomplicated implantation	43	10.1	No
2	46	Mechanical	AV	28	With Bentall-graft. Postoperative haematoma requiring re-thoracotomy and re-suturing of the graft	9	7.8	No
3	65	Mechanical	AV	645	Uncomplicated implantation	71	10.5	No
4	84	Biological	AV	21	Uncomplicated implantation	154	11.7	No
5	52	Mechanical	AV	922	Postoperative pneumothorax. No complications of the valve	184	12.7	No
6	83	Biological	AV	63	Uncomplicated implantation. CABG in same session	10	7.5	No
7	71	Biological	AV	150	Uncomplicated implantation	89	11.1	No
8	57	Mechanical	AV	2694	Uncomplicated implantation	35	7.8	No
9	49	Biological	AV	1769	With Bentall-graft. Uncomplicated implantation	35	11.5	Yes
10	75	Biological	AV	143	Postoperative pneumothorax. No complications of the valve. CABG in same session.	43	7.6	Yes
11	78	Biological	AV	101	Uncomplicated implantation. CABG in same session	2	4.1	Yes
12	23	Biological	PV	2501	Uncomplicated implantation	43	8.2	Yes
13	68	Biological	AV	662	Uncomplicated implantation	30	9.5	Yes
				710		8	7.9	Yes

Patient	PVE	FDG PET/CT visual interpretation		SUVmax		TBR	
		Standard	Late	Standard	Late	Standard	Late
1	No	True negative	False positive	3.37	3.92	1.57	3.21
2	No	False positive	False positive	5.38	6.30	2.96	5.63
3	No	True negative	False positive	3.33	3.23	1.71	2.36
4	No	True negative	True negative	2.68	2.39	2.14	2.60
5	No	True negative	True negative	2.38	2.26	1.71	2.31
6	No	True negative	False positive	3.01	3.74	2.39	3.43
7	No	True negative	False positive	3.31	4.64	1.91	4.22
8	No	True negative	True negative	3.58	4.11	2.29	3.95
9	Yes	True positive	True positive	4.83	6.76	3.45	7.95
10	Yes	True positive	True positive	4.27	4.51	3.26	4.65
11	Yes	True positive	True positive	3.23	3.13	1.78	3.16
12	Yes	True positive	True positive	3.99	3.88	3.05	4.26
13	Yes	True positive	True positive	6.16	7.92	5.01	8.43
	Yes	False negative	True positive	3.20	3.40	2.41	3.66

For the group as a whole, late images had significantly higher TBR values compared to the standard images, due mostly to a decrease in measured activity in the blood pool (SUVmax PHV standard 3.77 ± 1.06 vs. late 4.30 ± 1.64, *P* = 0.02; TBR standard 2.55 ± 0.93 vs. late 4.27 ± 1.89, *P* = 0.0001).

Contingency tables for visual interpretation of standard and late images are shown in Table 2. Sensitivity, specificity, positive predictive value and negative predictive value were 83%, 88%, 83%, and 88% respectively for the standard images and 100%, 38%, 55%, and 100%, respectively, for late images. Although late images correctly identified the single false negative result in the standard images, false positive results increased from 1 scan (7%) to 5 scans (36%).

**Table 2:** Contingency tables of the visual analysis

	Standard		Late	
	PET/CT+	PET/CT−	PET/CT+	PET/CT−
PVE+	5	1	6	0
PVE−	1	7	5	3

**Figure 1:**
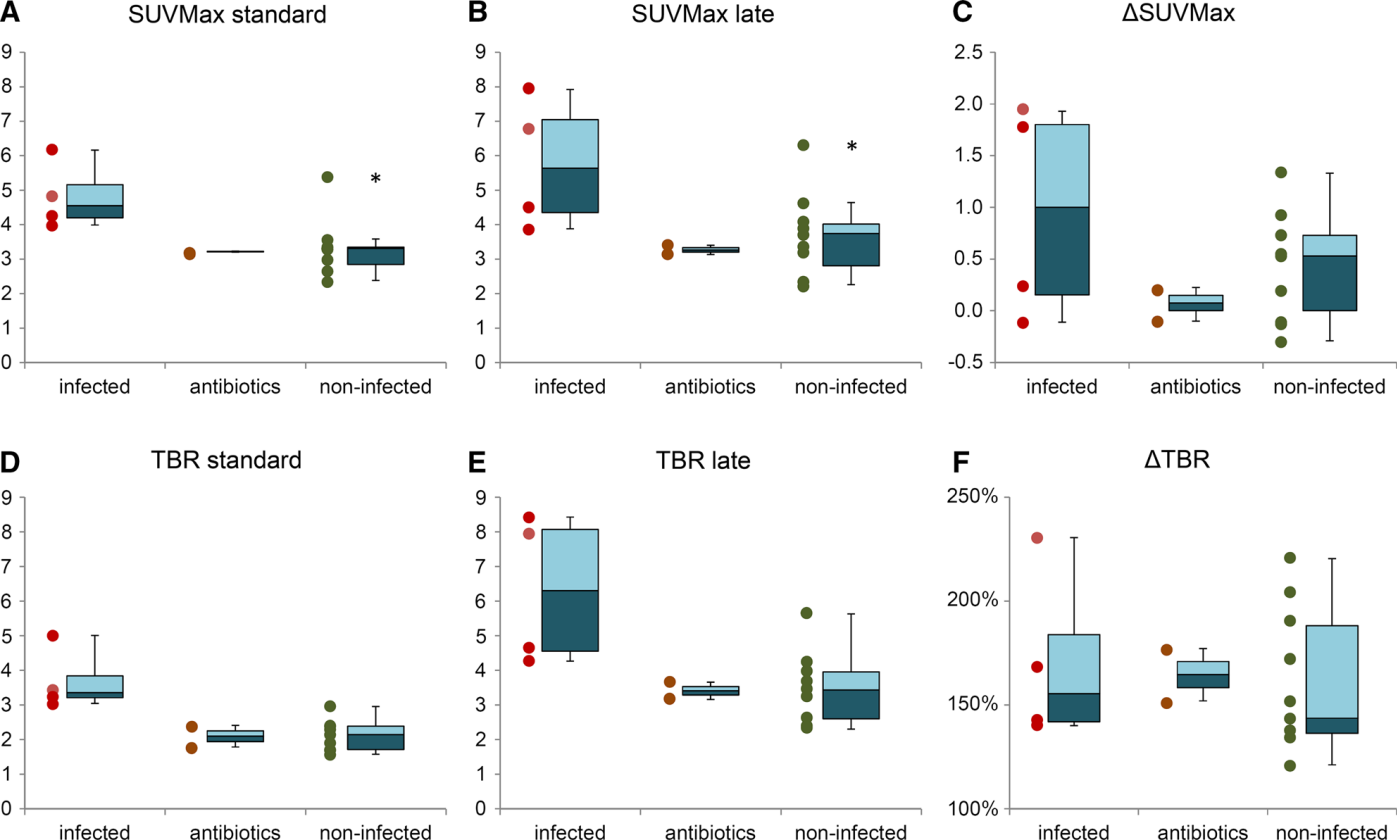
Semi-quantitative measurements. Values for standard SUVmax (**A**), late SUVmax (**B**), changes in SUVmax from standard to late (ΔSUVmax, **C**) as well as standard and late Target-to-background ratios (TBR) and changes in TBR (**D**, **E**, and **F** respectively) with *boxplot* representations of median values, interquartile ranges, and total ranges. * Statistical outlier

Standard SUVmax in the region of the PHV was higher in patients with PVE (median 4.13, interquartile range [IQR] 3.42-4.69) than in those without (median 3.32, IQR 2.93-3.42), but not significantly so (*P* = 0.12). The same was true for late SUVmax, but with even more overlap between the values (PVE median 4.20, IQR 3.52-6.20; non-PVE median 3.83, IQR 3.02-4.24. *P* = 0.23). In two scans of patients with PVE, the SUVs were comparatively low, probably due to effective antibiotic therapy at the time of imaging (based on normalized laboratory parameters and abated symptoms); when these scans were excluded from the analysis, standard SUVmax differed significantly (*P* = 0.02), while the difference in late SUVmax was still only trending towards significance (*P* = 0.07). When patients under adequate antibiotic therapy were regarded as a separate group, their values were more comparable to those in the disease-free group (Figure 1).

Only the TBR at the standard acquisition time was significantly different between infected and uninfected PHVs (*P* = 0.027), with sensitivity, specificity, positive predictive value, and negative predictive value of 83%, 88%, 83%, and 88%, respectively, at a threshold of 2.4 equalling the visual interpretation. Comparison of receiver operating characteristic curves showed a greater area under the curve for standard TBR (0.87) compared to late TBR (0.79), although the difference was not statistically significant (*P* = 0.22).

## Discussion

With the aim to improve our imaging protocol in patients with suspected PVE, according to the IDEAL criteria, we compared standard and late acquisition of FDG PET/CT images in suspected PVE in a small cohort of patients. The most important finding of our study was that delayed images did not compare favorably to standard images, as delayed images were more prone to false positive results. Changes in SUVmax and TBR between standard and late images showed great variation in both the PVE group and the non-PVE group, and almost complete overlap between the two groups. Although delayed images did show a higher contrast between target and background, due mostly to a decrease in activity in the blood pool, this higher contrast did not lead to better differentiation between infected and uninfected PHVs.

These findings may indicate that even in uninfected PHVs, a variable amount of sterile inflammation, likely a mild foreign body reaction, is present which may be indistinguishable from indolent or low-grade infection. As no histological data were retrieved from any non-infected PHVs, we cannot be certain about the underlying cause or why some PHVs show more inflammation than others.

**Figure 2:**
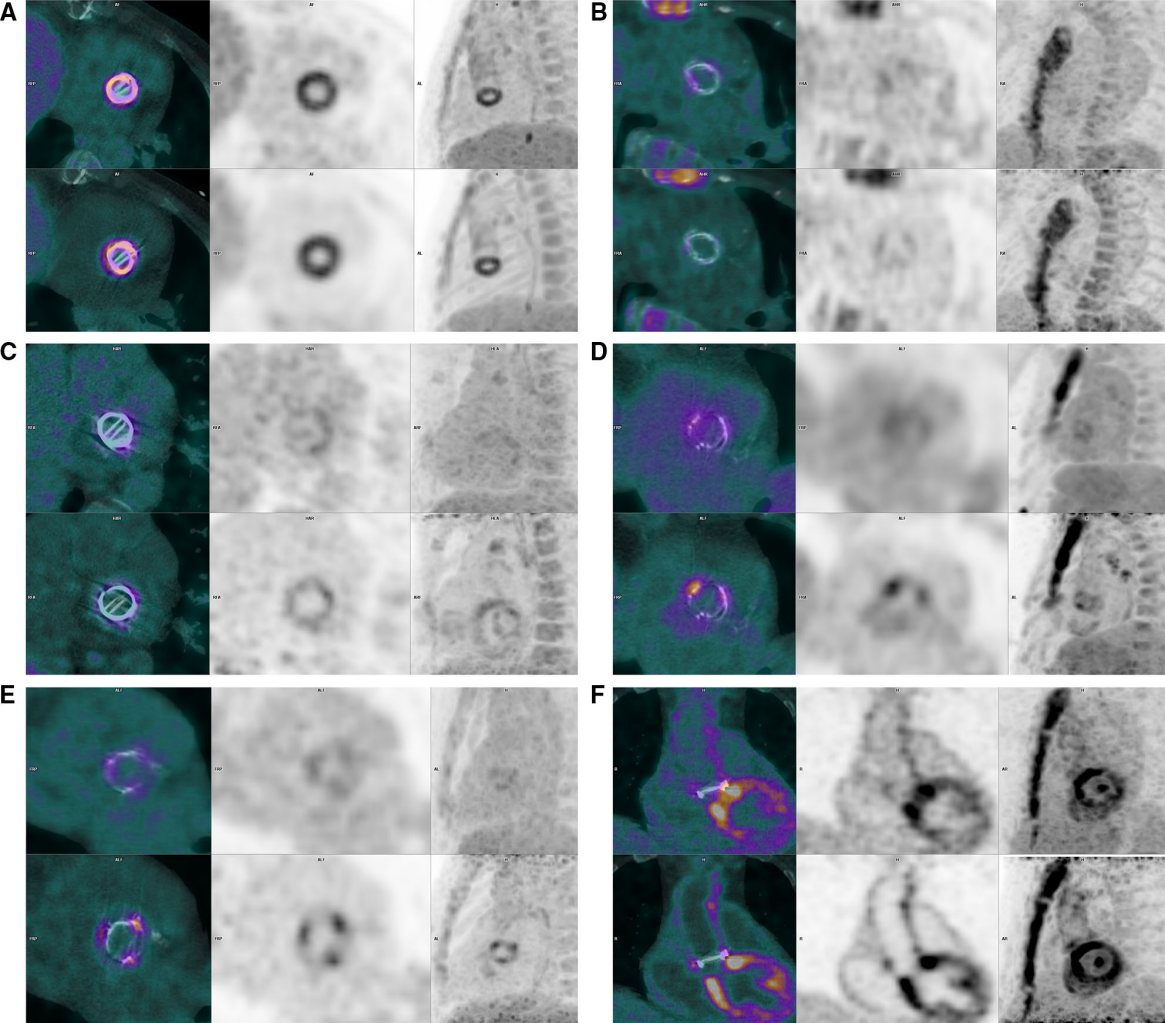
Examples of standard (*top row*) and late (*bottom row*) FDG PET/CT images in six patients. **A** True positive uptake surrounding a mechanical aortic PHV in both phases. **B** True negative images with hardly any uptake near the biological aortic PHV in either phase. **C** True negative standard images with minimal uptake surrounding the mechanical mitral PHV, clearly delineated in the late image. **D** True positive late phase with focal uptake near the strut of the biological aortic PHV near the right coronary that is not apparent on standard images. Note accumulation of uptake in mediastinal lymph nodes. **E** False positive late images with exaggerated uptake near all struts of a biological aortic PHV with normal uptake on standard images. **F** False positive uptake in both phases surrounding the prosthesis after semi-recent Bentall procedure complicated by haematoma. Note the unsuppressed physiological uptake in the myocardium

In two patients, standard FDG PET/CT showed very low uptake of FDG around the PHV, even though both patients were diagnosed with PVE (Table 1, patient 11 and second scan of patient 13). However, both patients were free of signs and symptoms and had normalized laboratory parameters for infection after antibiotic treatment at the time of PET/CT acquisition. It is likely that FDG PET/CT showed the true effect of therapy in these patients, as the subsequent and final diagnosis of PVE was made at surgery after signs, symptoms, and laboratory values had increased following cessation of antibiotic therapy. As we have described earlier,^18^ an initial response to antibiotic therapy does not necessarily imply eradication of the causative pathogen. In one of these two patients, the delayed images did show increased uptake where the standard images did not, but this also occurred in the late images of several patients without PVE and should therefore be interpreted with caution (Figure 2).

Conversely, standard PET/CT was false positive in one patient, with high SUVmax and TBR values compared to the other values in the uninfected group (Table 1, patient 2). This scan was performed 4 weeks after a Bentall procedure complicated by bleeding, which necessitated a re-sternotomy to drain 400 ml of haematoma and re-suturing of the graft. It is likely that imaging relatively shortly after such a complicated procedure visualized sterile inflammation as part of the immune response to the haematoma. Delayed images did not contribute to the differentiation of sterile inflammation from infection.

These cases stress the importance of being well informed about the patient history before interpreting FDG PET/CT in patients with suspected PVE, as many factors including details about the implantation, current signs and symptoms, medication, and changes in inflammatory laboratory parameters over time may considerably influence the interpretation of findings.

In the other four scans with negative standard and positive late images, two patients had positive blood cultures with *Staph. aureus* and were receiving antibiotic treatment at the time of imaging, which could potentially have led to false negative interpretation of the standard images. However, as both cases had well-defined infectious foci elsewhere (presternal wound infection in one and infected knee prosthesis in the other) which were clearly visible on FDG PET/CT and responded well to local therapy, PVE was not diagnosed clinically and we believe it is more likely that the late images were false positive. The other two patients had negative blood cultures and were not receiving antibiotics, further corroborating that false positive late images in the absence of PVE do occur.

Current opinion on the timeframe after surgery after which FDG PET/CT can be reliably performed varies, with indications that sterile postoperative inflammation may persist as long as two months after surgery.^10^ In our cohort, true negative images were obtained as early as three weeks after surgery whereas false positive late images occurred more than 13 years after implantation. In our experience, scans may be performed as early as a few weeks after uncomplicated surgery. If complications occurred during or after surgery (as in the Bentall procedure described above) sterile inflammation may persist for longer.

In this study, only the standard TBR showed a statistically significant difference between infected and uninfected PHVs; standard SUVmax trended towards but did not reach significance, likely due to the small number of patients and the confounding factors mentioned above.

Defining threshold values on a cohort as small as ours should be done with extreme caution, but TBR at the standard acquisition time at a threshold of 2.4 did perform as well as visual analysis, with equal sensitivity, specificity, and positive and negative predictive values. Perhaps of greater clinical value is the finding that all scans with TBR >3.0 were infected. Still, some confounders such as surgical adhesive and lipomatous hypertrophy of the interatrial septum show intense uptake of FDG^17^ and would probably score above this threshold, and our data show that scans performed under adequate antibiotic therapy will be underestimated.

When comparing our data to the aforementioned earlier reports, the case presented by Calderella et al.^13^ fits with our findings, basically mirroring the one case in our series that was false negative on standard images and true positive on late images (Figure 2D). However, our data suggest that applying this approach to every negative standard FDG PET/CT would lead to an unwarranted number of false positive late scans. Leccisotti et al.^14^ found added value for late images in diagnosing cardiac implantable electronic device lead infection, which is likely to have a different presentation compared to PVE. False positive findings did occur for lead infection, but did not increase on late images, as opposed to our results in patients with PVE. Therefore, these findings are not necessarily at odds with each other. It may very well be that delayed images have added value in diagnosing lead infection because of the high false negative rate on standard images and the apparent lack of physiologically increased uptake due to sterile inflammation, as opposed to imaging in PVE.

Our findings are limited by the small amount of patients included in a single center, and the retrospective nature of the data. Ideally, our findings should be corroborated by a prospective study powered for statistical analysis. In these early stages of development of FDG PET/CT protocols specifically applicable to PVE diagnosis, we essentially find ourselves moving from stage 1 of the IDEAL framework (Idea) to stage 2a (Development) where the details about a new procedure become more defined.^15^ Regardless of its limitations, our data show a clinically important risk of false positive PVE findings if late images at 150 minutes post injection are allowed to guide treatment decisions, which should be taken into account in possible future studies and their design. It is possible that the ideal time point for FDG PET/CT imaging in PVE lies somewhere between our two chosen time points.

## New Knowledge Gained

Based on our current data, we cannot recommend the use of delayed FDG PET/CT imaging in PVE, whether as substitution for or as an adjunct to standard images, as we believe the risk of false positive interpretation is too high in either scenario.

## Conclusion

Delayed imaging at 150 minutes post injection does not seem to improve the interpretation of FDG PET/CT in PVE as it seems prone to false positive findings. Imaging at the standard oncology protocol acquisition time outperformed delayed imaging both in visual and semi-quantitative analysis.

## Electronic supplementary material

Below is the link to the electronic supplementary material.

Supplementary material 1 (PPTX 638 kb)

## Abbreviations

CTA: Computed tomography angiography
FDG: Fluorine-18 fluorodeoxyglucose
IQR: Interquartile range
MBq: Megabecquerel
PET: Positron emission tomography
PHV: Prosthetic heart valve
PVE: Prosthetic heart valve endocarditis
SUV: Standardized uptake value
TBR: Target-to-background ratio
VOI: Volume of interest
