# Supplementary material for: Dual-time-point FDG PET/CT imaging in prosthetic heart valve endocarditis
Source: J Nucl Cardiol. 2017 May 4;25(6):1960–7. doi: 10.1007/s12350-017-0902-3 (PMC6280957; doi:10.1007/s12350-017-0902-3)
Supplement: Supplementary file 1 — Supplementary material 1 (PPTX 638 kb) [file 12350_2017_902_MOESM1_ESM.pptx]

## Slide 1
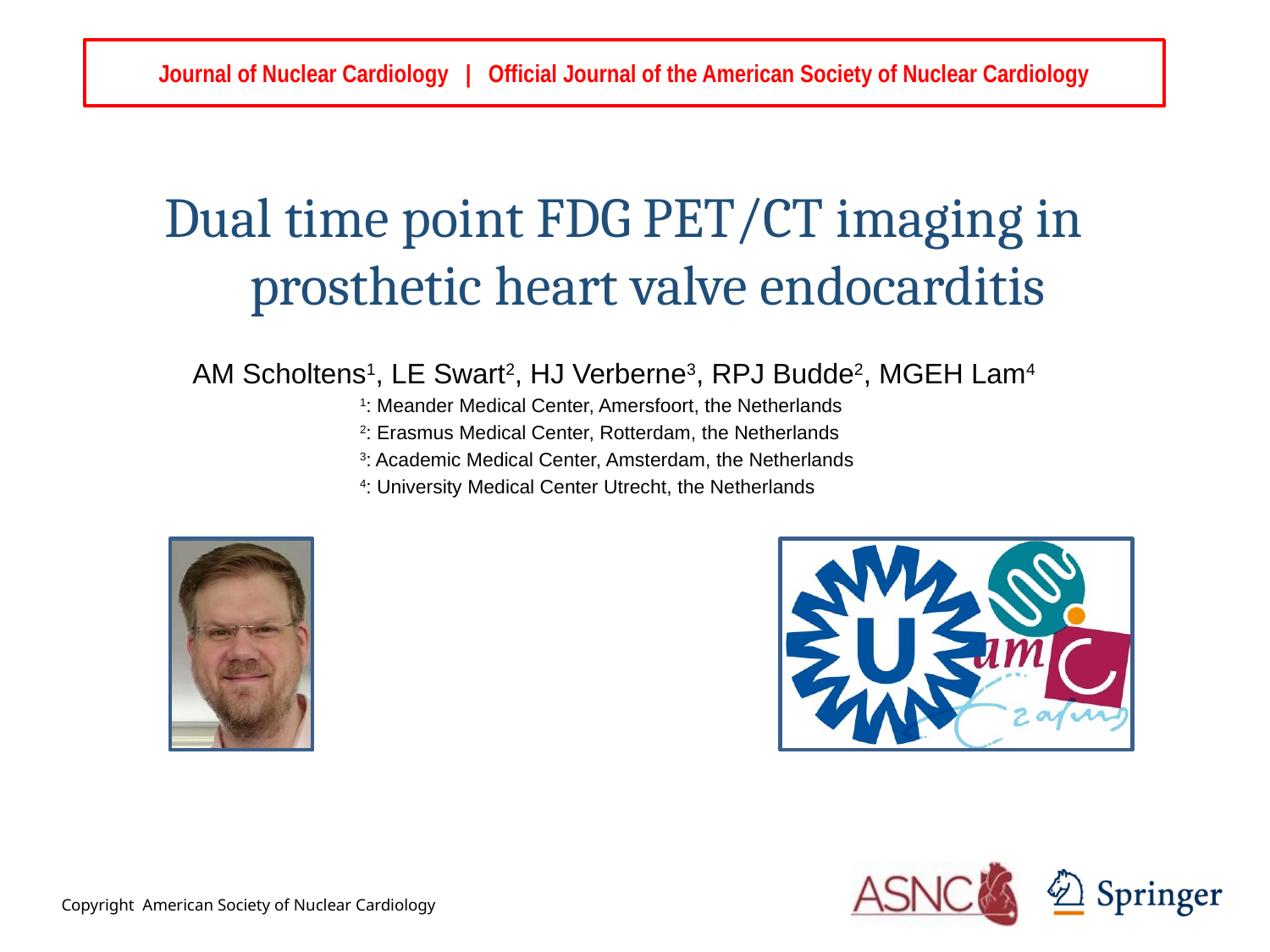

Journal of Nuclear Cardiology | Official Journal of the American Society of Nuclear Cardiology
# Dual time point FDG PET/CT imaging in prosthetic heart valve endocarditis
AM Scholtens1, LE Swart2, HJ Verberne3, RPJ Budde2, MGEH Lam4
1: Meander Medical Center, Amersfoort, the Netherlands
2: Erasmus Medical Center, Rotterdam, the Netherlands
3: Academic Medical Center, Amsterdam, the Netherlands
4: University Medical Center Utrecht, the Netherlands
Copyright American Society of Nuclear Cardiology

## Slide 2
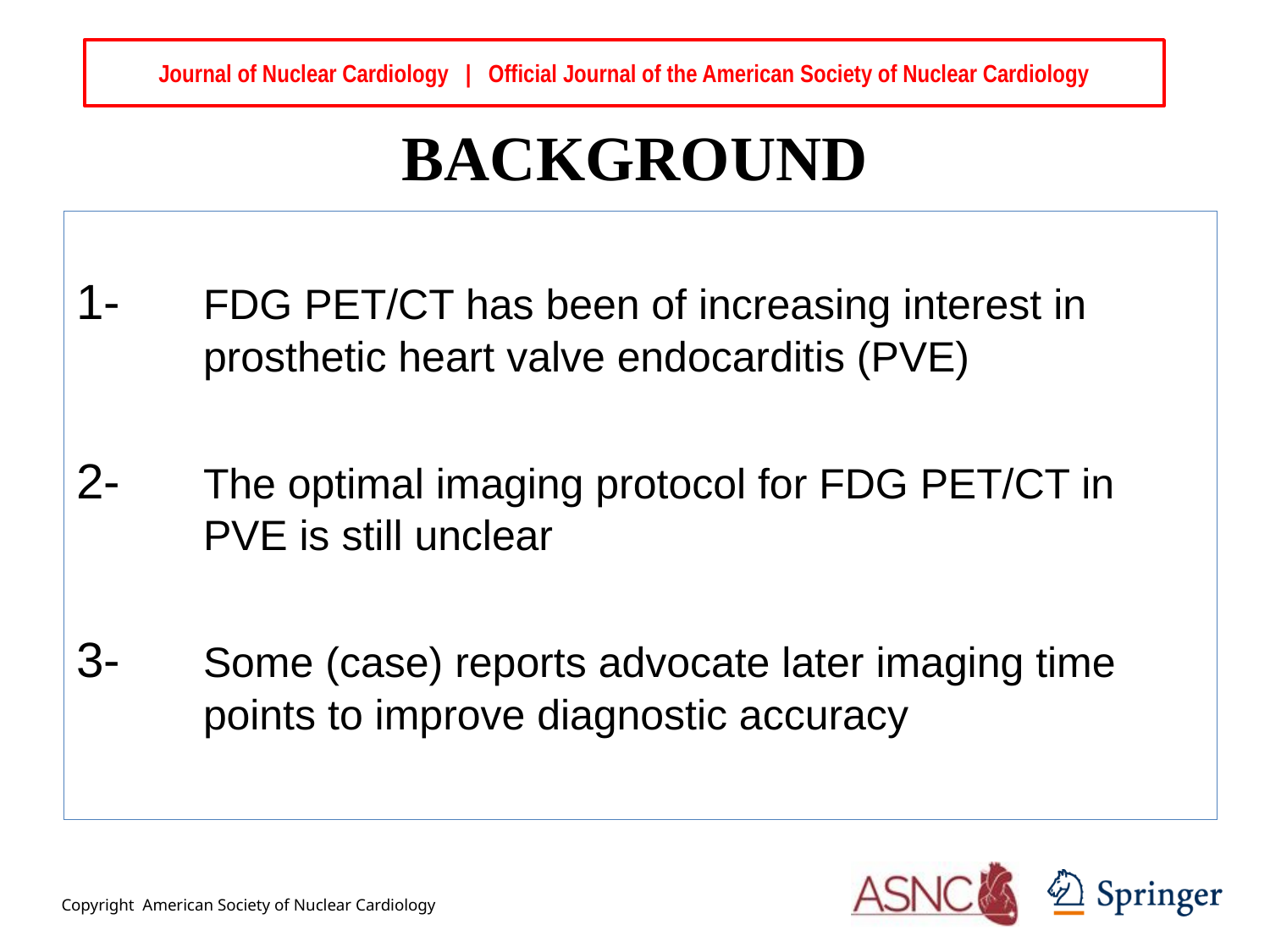

Journal of Nuclear Cardiology | Official Journal of the American Society of Nuclear Cardiology
# BACKGROUND
1-	FDG PET/CT has been of increasing interest in 	prosthetic heart valve endocarditis (PVE)
2- 	The optimal imaging protocol for FDG PET/CT in 	PVE is still unclear
3-	Some (case) reports advocate later imaging time 	points to improve diagnostic accuracy
Copyright American Society of Nuclear Cardiology

## Slide 3
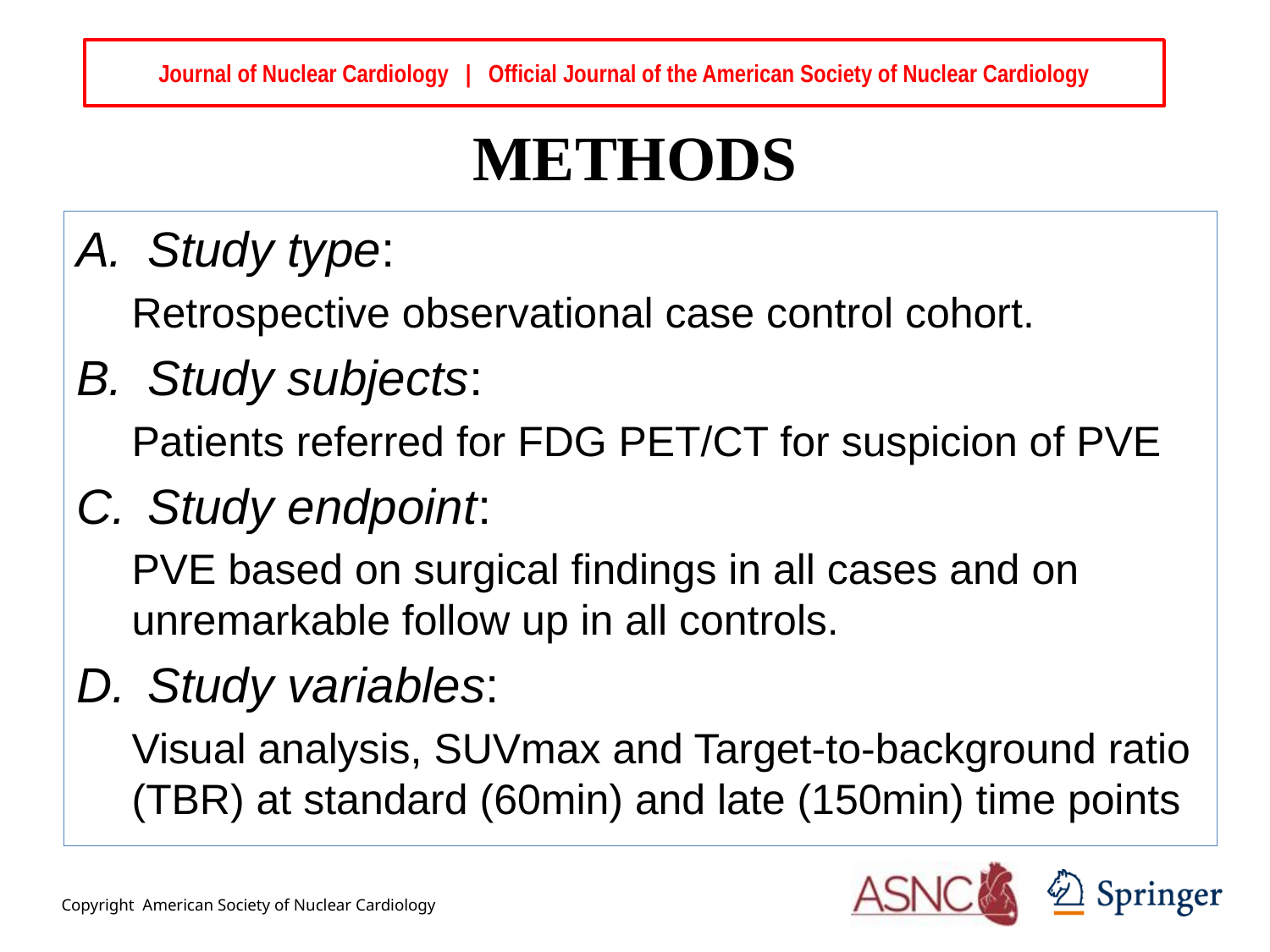

Journal of Nuclear Cardiology | Official Journal of the American Society of Nuclear Cardiology
# METHODS
Study type:
Retrospective observational case control cohort.
Study subjects:
Patients referred for FDG PET/CT for suspicion of PVE
Study endpoint:
PVE based on surgical findings in all cases and on unremarkable follow up in all controls.
Study variables:
Visual analysis, SUVmax and Target-to-background ratio (TBR) at standard (60min) and late (150min) time points
Copyright American Society of Nuclear Cardiology

## Slide 4
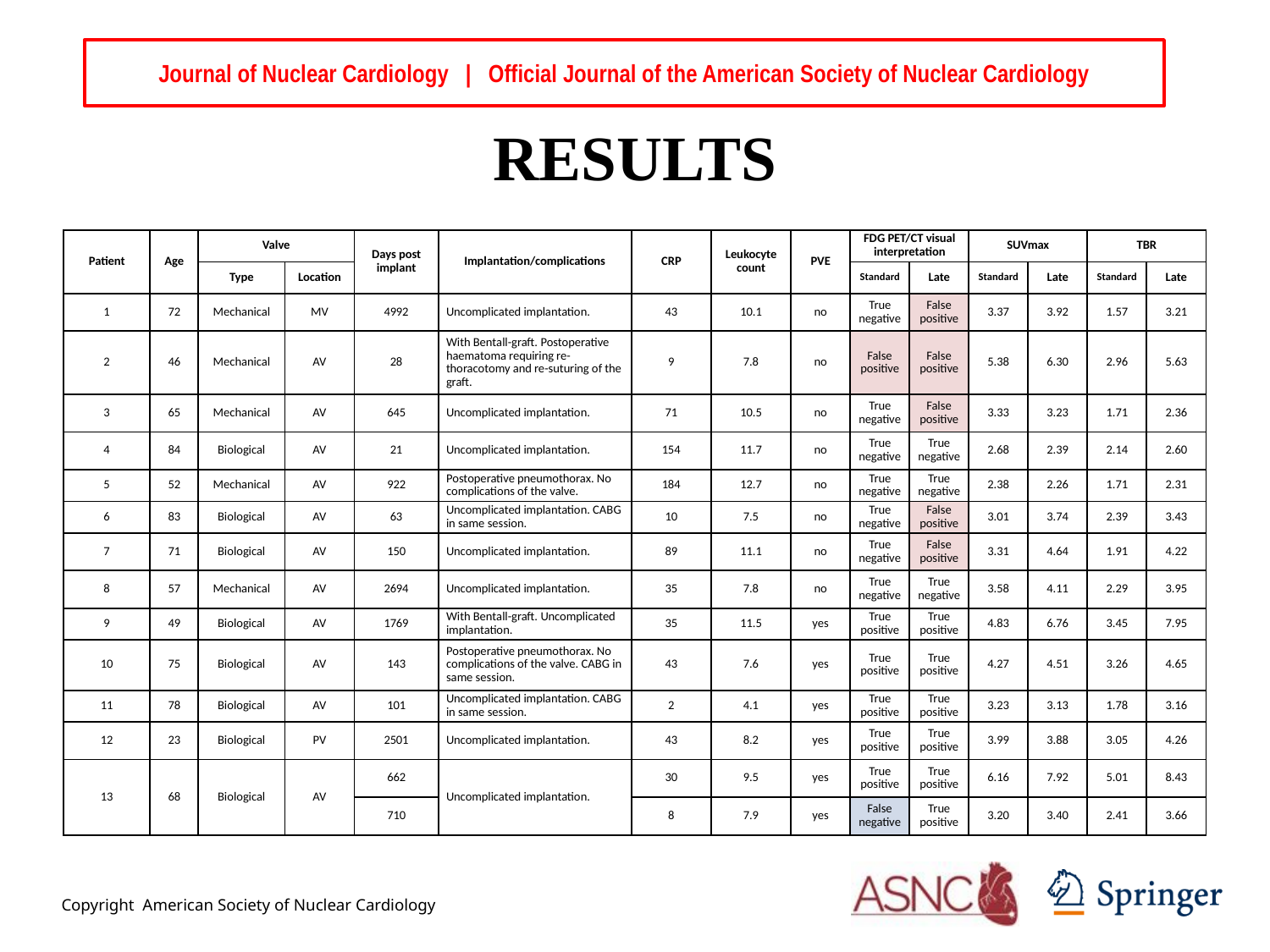

Journal of Nuclear Cardiology | Official Journal of the American Society of Nuclear Cardiology
# RESULTS
| Patient | Age | Valve | | Days post implant | Implantation/complications | CRP | Leukocyte count | PVE | FDG PET/CT visual interpretation | | SUVmax | | TBR | |
| --- | --- | --- | --- | --- | --- | --- | --- | --- | --- | --- | --- | --- | --- | --- |
| | | Type | Location | | | | | | Standard | Late | Standard | Late | Standard | Late |
| 1 | 72 | Mechanical | MV | 4992 | Uncomplicated implantation. | 43 | 10.1 | no | True negative | False positive | 3.37 | 3.92 | 1.57 | 3.21 |
| 2 | 46 | Mechanical | AV | 28 | With Bentall-graft. Postoperative haematoma requiring re-thoracotomy and re-suturing of the graft. | 9 | 7.8 | no | False positive | False positive | 5.38 | 6.30 | 2.96 | 5.63 |
| 3 | 65 | Mechanical | AV | 645 | Uncomplicated implantation. | 71 | 10.5 | no | True negative | False positive | 3.33 | 3.23 | 1.71 | 2.36 |
| 4 | 84 | Biological | AV | 21 | Uncomplicated implantation. | 154 | 11.7 | no | True negative | True negative | 2.68 | 2.39 | 2.14 | 2.60 |
| 5 | 52 | Mechanical | AV | 922 | Postoperative pneumothorax. No complications of the valve. | 184 | 12.7 | no | True negative | True negative | 2.38 | 2.26 | 1.71 | 2.31 |
| 6 | 83 | Biological | AV | 63 | Uncomplicated implantation. CABG in same session. | 10 | 7.5 | no | True negative | False positive | 3.01 | 3.74 | 2.39 | 3.43 |
| 7 | 71 | Biological | AV | 150 | Uncomplicated implantation. | 89 | 11.1 | no | True negative | False positive | 3.31 | 4.64 | 1.91 | 4.22 |
| 8 | 57 | Mechanical | AV | 2694 | Uncomplicated implantation. | 35 | 7.8 | no | True negative | True negative | 3.58 | 4.11 | 2.29 | 3.95 |
| 9 | 49 | Biological | AV | 1769 | With Bentall-graft. Uncomplicated implantation. | 35 | 11.5 | yes | True positive | True positive | 4.83 | 6.76 | 3.45 | 7.95 |
| 10 | 75 | Biological | AV | 143 | Postoperative pneumothorax. No complications of the valve. CABG in same session. | 43 | 7.6 | yes | True positive | True positive | 4.27 | 4.51 | 3.26 | 4.65 |
| 11 | 78 | Biological | AV | 101 | Uncomplicated implantation. CABG in same session. | 2 | 4.1 | yes | True positive | True positive | 3.23 | 3.13 | 1.78 | 3.16 |
| 12 | 23 | Biological | PV | 2501 | Uncomplicated implantation. | 43 | 8.2 | yes | True positive | True positive | 3.99 | 3.88 | 3.05 | 4.26 |
| 13 | 68 | Biological | AV | 662 | Uncomplicated implantation. | 30 | 9.5 | yes | True positive | True positive | 6.16 | 7.92 | 5.01 | 8.43 |
| | | | | 710 | | 8 | 7.9 | yes | False negative | True positive | 3.20 | 3.40 | 2.41 | 3.66 |
Copyright American Society of Nuclear Cardiology

## Slide 5
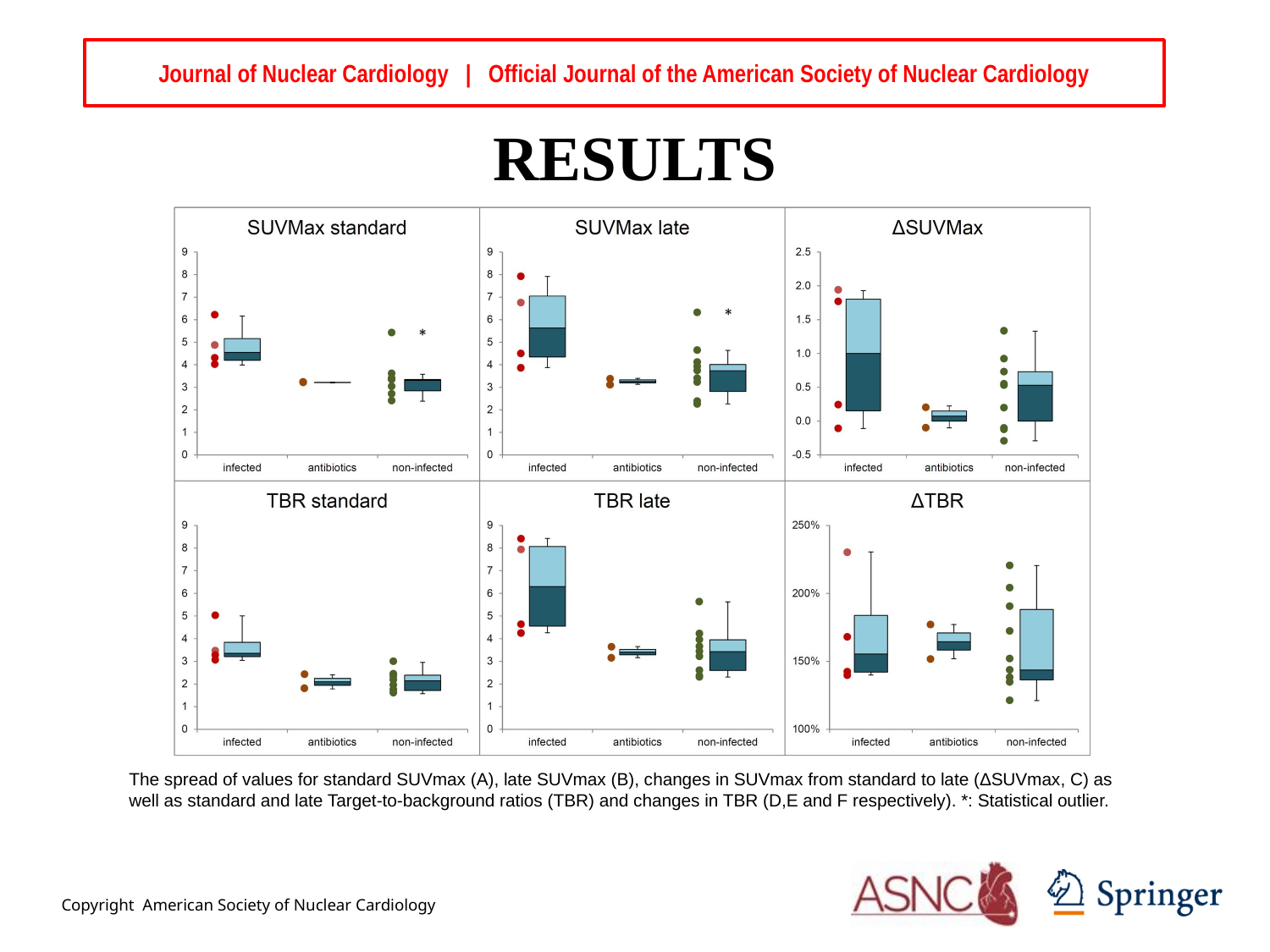

Journal of Nuclear Cardiology | Official Journal of the American Society of Nuclear Cardiology
# RESULTS
The spread of values for standard SUVmax (A), late SUVmax (B), changes in SUVmax from standard to late (ΔSUVmax, C) as well as standard and late Target-to-background ratios (TBR) and changes in TBR (D,E and F respectively). *: Statistical outlier.
Copyright American Society of Nuclear Cardiology

## Slide 6
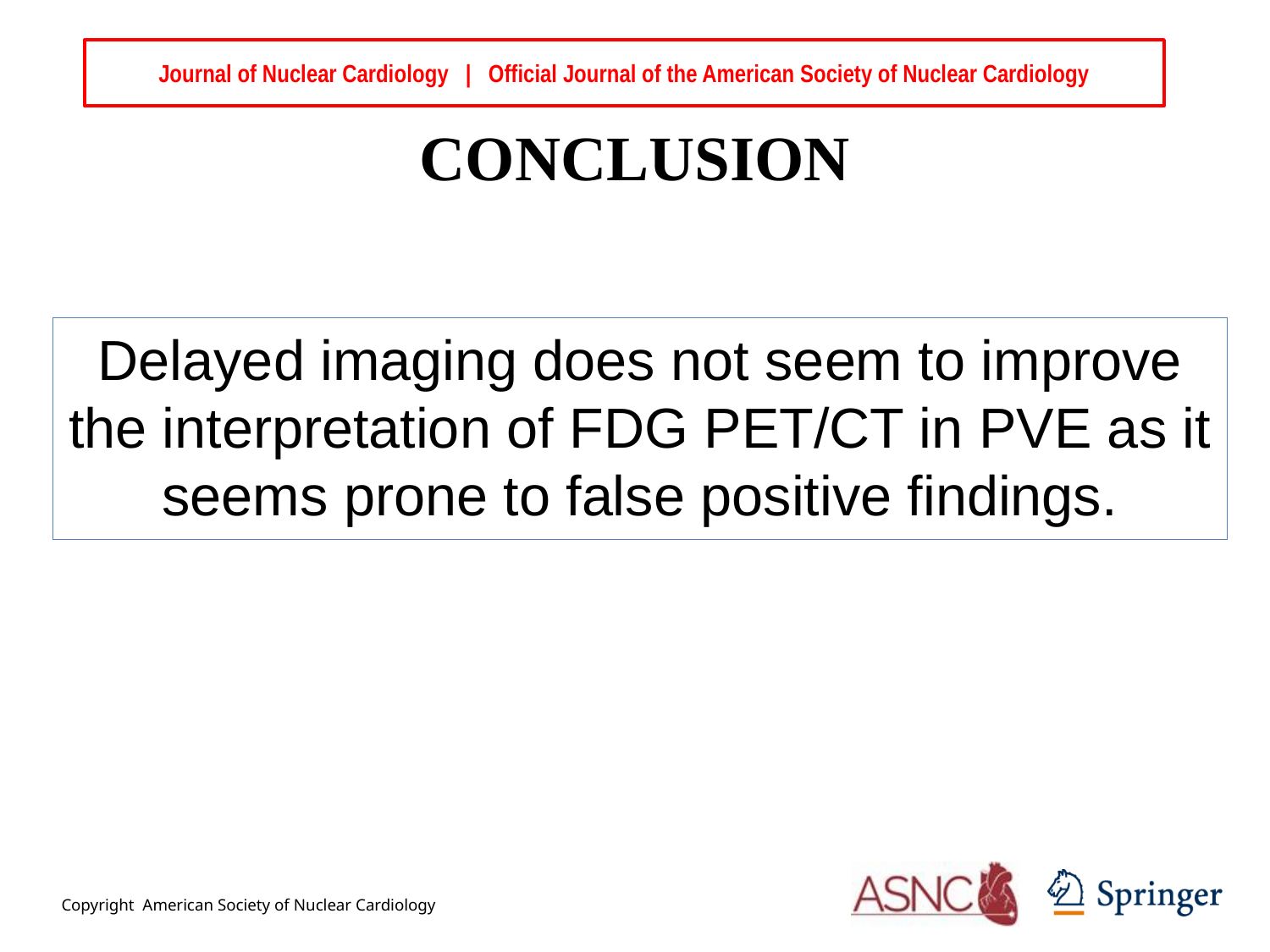

Journal of Nuclear Cardiology | Official Journal of the American Society of Nuclear Cardiology
# CONCLUSION
Delayed imaging does not seem to improve the interpretation of FDG PET/CT in PVE as it seems prone to false positive findings.
Copyright American Society of Nuclear Cardiology
